# The predictive value of abnormal electrocardiogram for patent foramen ovale: A retrospective study

**DOI:** 10.1002/clc.24133

**Published:** 2023-09-04

**Authors:** Peng Jin, Piqi Jiao, Juan Feng, Liang Shi, Ling Ma

**Affiliations:** ^1^ Department of Cardiovascular Medicine the 940th Hospital of the Joint Logistics Support Force of the Chinese People's Liberation Army Lanzhou China

**Keywords:** foramen ovale, patent, electrocardiography, crochetage R wave

## Abstract

**Background and Hypothesis:**

The objective of this study was to identify the characteristics of electrocardiogram (ECG) in adult patients with patent foramen ovale (PFO) and to analyze the predictive value of the characteristics of ECG for PFO in adult patients.

**Methods:**

Retrospectively, 267 patients who had undergone ECG, transthoracic echocardiography (TTE), transesophageal echocardiography (TEE) with agitated saline contrast echocardiography in our hospital, were recruited continuously from January 2021 to March 2023. Electrocardiographs were analyzed to investigate the presence of right bundle branch block (RBBB) and crochetage R wave.

**Results:**

The ratio of crochetage R wave in inferior leads in patients with PFO was 45.3% and 21.2% without PFO. There were 17 (6.4%) patients with coexistence of crochetage R wave and RBBB, including 13 (6.5%) patients with PFO and four (6.1%) patients without PFO. The accuracies of TTE, crochetage R wave, and RBBB were 0.637, 0.535, and 0.314, respectively. A combination of crochetage R wave and RBBB demonstrated a sensitivity of 0.507 and a specificity of 0.758. When TTE, crochetage R wave, and RBBB were combined, the accuracy, sensitivity, specificity, positive predictive value, and negative predictive value were 0.712, 0.801, 0.439, 0.813, and 0.420, respectively. Logistic regression analysis revealed a correlation between PFO and the presence of crochetage R wave (odds ratio [OR]: 3.073, 95% confidence interval [CI]: 1.601–5.899, *p* < 0.001), and also a combination between crochetage R wave and RBBB (OR: 3.220, 95% CI: 1.720–6.028, *p* < 0.001).

**Conclusions:**

Crochetage R wave in ECG was associated with PFO. Crochetage R wave, especially combined with RBBB and TTE, may be helpful in the early detection of patients with PFO.

## Graphical abstract

Crochetage R wave in inferior leads of electrocardiogram was associated with patent foramen ovale. The accuracy of crochetage R wave was 53.5% (sensitivity: 45.3%; specificity: 78.8%).

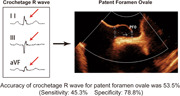

## 1 INTRODUCTION

Patent foramen ovale (PFO) is a congenital cardiac abnormality usually incidentally found. A PFO is seen in about 25% of the general population and may therefore coexist by chance in a patient with an unexplained left circulation embolism due to right to left shunt (RLS).^1^, ^2^ PFO is implicated in the pathogenesis of a number of medical conditions such as cryptogenic stroke,^1^, ^2^, ^3^ decompression sickness,^4^ secondary migraine headache,^5^ arterial deoxygenation, and platypnea‐orthodeoxia.^6^, ^7^ Early detection of PFO is of major importance for thromboembolic events.

Transthoracic echocardiography (TTE) is often unable to clearly display the anatomical structure of PFO, while intermittent low‐speed RLS Doppler signals are less likely to be detected.^8^ Transesophageal echocardiography (TEE) with the addition of agitated saline contrast (ASC) and appropriately performed provocative maneuvers is considered the gold‐standard imaging modality for identification of an intracardiac shunt such as PFO.^9^ However, it is not easily accepted due to its invasiveness and lack of convenience. Especially the Valsalva maneuver is difficult to perform for patients undergoing TEE. Hence, it is important to find indicators that will guide to early diagnosis of PFO with noninvasiveness and convenience. Abnormal electrocardiogram (ECG) signs of right bundle branch block (RBBB)^10^ or a notch on R wave in inferior ECG leads (II, III, augmented vector foot [aVF])^11^, ^12^ may offer some clues that indicate PFO.

Our aims in this study were to identify the characteristics of ECG such as RBBB or a notch on R wave in inferior ECG leads in adult patients with PFO diagnosed by TEE with ASC echocardiography, and to analyze the predictive value of the characteristics of ECG for PFO in adult patients.

## 2 METHODS

### 2.1 Ethics

This study was registered on the China Clinical Trial Registry website: https://www.chictr.org.cn (ChiCTR2300070692). The study was approved by the Institutional Clinical Ethics Committee of the 940th Hospital of the Joint Logistics Support Force of the Chinese People's Liberation Army and performed in accordance with the CONSORT 2010 guidelines and in accordance with the Declaration of Helsinki (1964). All patients or their relatives provided written informed consent.

### 2.2 Patient population

We completed a retrospective, cross‐sectional study on 267 adult patients. All patients who had undergone ECG, TTE, TEE, and ASC echocardiography in our hospital were recruited continuously during the period of January 2021 until March 2023. Patients with coronary heart disease, cardiomyopathy, valvular heart disease, severe respiratory diseases, or tumors were excluded from the study. Patients younger than 18 years old were excluded. Of all the patients, 201 were with PFO diagnosed by TEE and ASC echocardiography, whereas 66 patients were without PFO. The information related to the patients was collected from medical records.

### 2.3 ECG studies

**Figure 1:**
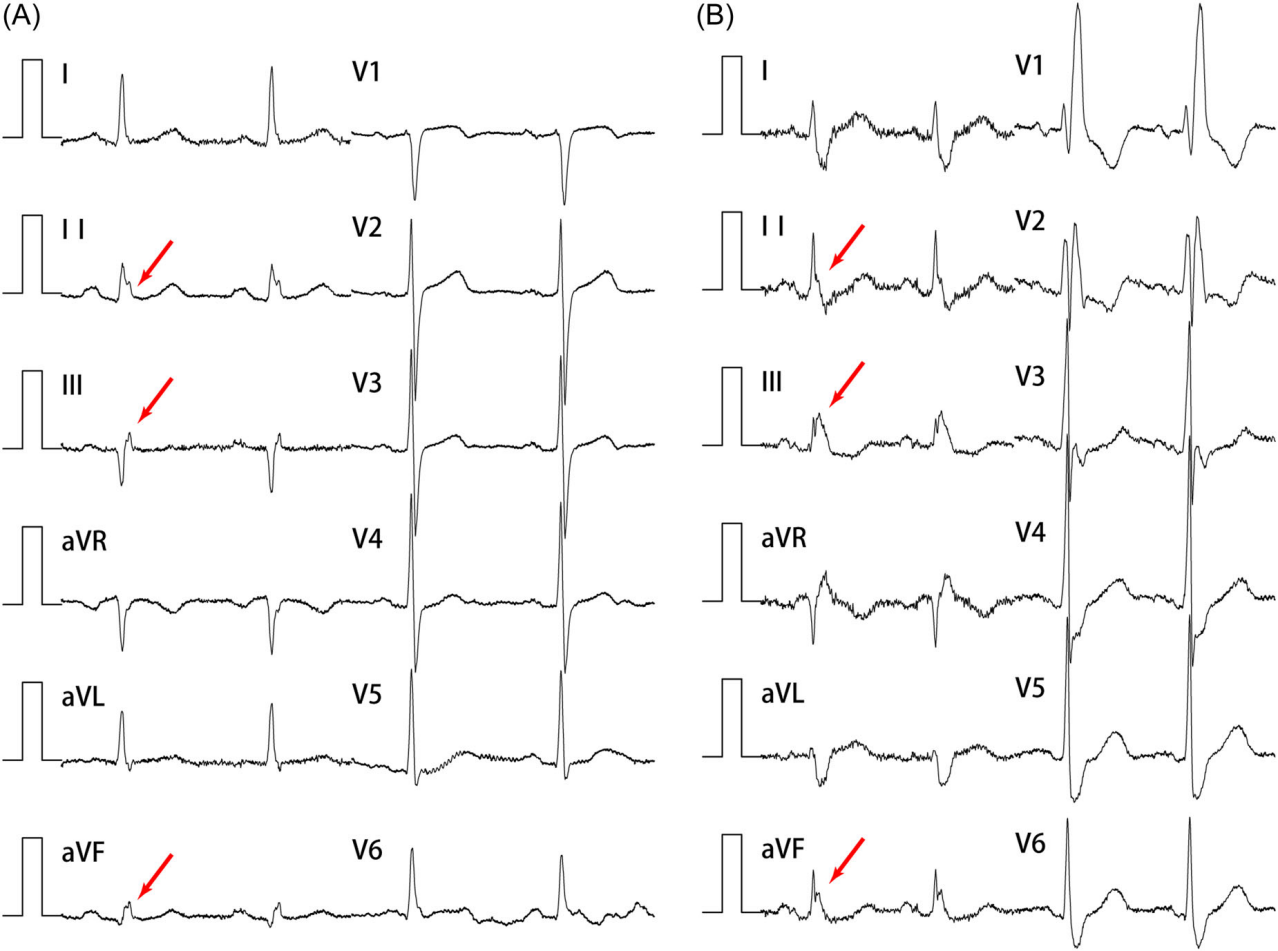
Crochetage R wave in inferior leads of electrocardiogram (A: a 58‐year‐old male patient with patent foramen ovale classified extensive right to left shunt; B: a 68‐year‐old male patient with patent foramen ovale classified moderate right to left shunt with complete right bundle branch block). aVF, augmented vector foot; aVL, augmented vector left; aVR, augmented vector right.

All patients had a standard 12‐lead ECG with a sensitivity of 10 mm/mV and paper speed of 25 mm/s using the MedEx MECG‐300 multilead ECG analysis system (MedEx). ECG result was analyzed carefully with respect to heart rhythm, heart rate, electric axis, and right ventricular repolarisation abnormalities. Special emphasis was given to analyzing disorders of impulse conduction, particularly the RBBB, which was defined as an RSR′ wave with R′ taller than R in leads V1 and V2, a greater duration of the S wave than the R wave in leads I and V6. In addition, a “crochetage” pattern in ECG, an M‐shaped bifid notch on the ascending branch, or on the zenith, of the R wave in inferior leads (II, III, aVF) (crochetage R wave) described by Heller et al.^11^ and Ay et al.^12^ was investigated (Figure 1). Analysis was performed by two examiners blinded for the study.

### 2.4 Echocardiographic studies

The TTE study was conducted by an experienced sonographer using the Philips EPIQ 7C imaging systems (Philips Medical Systems) equipped with an S5‐1 probe. TEE was performed using the same system fitted with a S7‐3t probe. Ten minutes before the TEE procedure, all patients received 2% lidocaine mucilage for oropharynx anesthesia. The probe was rotated within 30°–100° to clearly display the septum primum and septum secundum, as well as to obverse whether an opened PFO and RLS existed both in two‐ and three‐dimensional color Doppler ultrasonography.

ASC echocardiography studies were performed by injection of 4 mL of vitamin B6 and 6 mL of 5% sodium bicarbonate solution as a bolus without agitating into an antecubital vein at rest, and with Valsalva maneuver, as described by Zhao et al.^13^ The appearance of microbubbles in the left atrium within three to five cardiac beats after right atrial opacification is considered positive for the presence of an RLS considered to be the result of a PFO.^8^ The semiquantitative classification of PFO‐RLS was based on the maximum number of microbubbles presented in the left atrium on a still frame and was defined according to the following criteria: when no, 1–10 bubbles, 11–30 bubbles, and >30 bubbles (or left atrial opacity) were detected, the RLS was considered to be negative, mild, moderate, and extensive, respectively.^8^, ^13^, ^14^

### 2.5 Statistical analysis

The Kolmogorov–Smirnov test was used to assess the distribution of continuous variables. All continuous values were expressed as medians and interquartile ranges and all categorical variables were expressed as frequency and fractions. Comparison between continuous variables was made using Mann–Whitney *U* test, and for categorical variables, the *χ* ^2^ test was used. Multivariate analysis was used when more than two variables were compared. Diagnostic accuracy, sensitivity, specificity, positive predictive value (PPV), and negative predictive value (NPV) of TTE and ECG parameters were calculated, with 95% confidence interval (CI) based on the Clopper–Pearson exact method. Univariate logistic regression analysis was used to evaluate the independent associates of the risk of PFO. For all tests, a *p* value less than 0.05 was considered statistically significant. All statistical analyses were performed using SPSS software (version 20.0; IBM Inc.), and the Clopper–Pearson exact method was performed using STATA software (version 16.0; STATA Corp. LLC).

## 3 RESULTS

### 3.1 Patient characteristics

The clinical characteristics of patients are shown in Table 1. The study included 267 patients. There were 201 of them with PFO and 66 without PFO diagnosed by TEE and ASC echocardiography. Of all patients with PFO, 39 (19.4%) described it as mild, 84 (41.8%) as moderate, and 78 (38.8%) as extensive, according to the semiquantitative classification of PFO‐RLS. The ratio of crochetage R wave in inferior leads in patients with PFO (45.3%) was significantly increased compared to patients without PFO (21.2%) (*p* < 0.001). There were 17 (6.4%) patients with coexistence of crochetage R wave and RBBB, including 13 (6.5%) patients with PFO and four (6.1%) patients without PFO (*p* < 0.001). However, there was no significant difference in the ratio of RBBB between the patients with and without PFO. Also, there was no significant difference in the parameters of echocardiography, including left atrial longitudinal diameter, right atrial diameter, left ventricular end‐diastolic diameter, right ventricular end‐diastolic diameter, left ventricular ejection fraction, and left ventricular fractional shortening.

**Table 1:** Clinical characteristics of patients.

Variables	All (*n* = 267)	Without PFO (*n* = 66)	With PFO (*n* = 201)	*p* Value
Age (years)	53.1 (35.7, 60.8)	56.5 (52.0, 68.0)	51.0 (32.0, 59.0)	<0.001
Female gender (*n*, %)	100 (37.5)	27 (56.1)	63 (31.3)	<0.001
Smoking (*n*, %)	85 (31.8)	13 (19.7)	72 (35.8)	0.015
Drinking (*n*, %)	61 (22.8)	11 (16.7)	50 (24.9)	0.169
SBP (mm Hg)	127.6 (117.9, 140.8)	131.0 (121.8, 148.0)	126.0 (117.0, 139.0)	0.009
DBP (mm Hg)	81.1 (74.2, 87.9)	82.5 (75.8, 89.3)	81.0 (73.0, 87.5)	0.339
BMI	23.8 (21.7, 25.9)	25.0 (22.0, 26.0)	24.0 (22.0, 26.0)	0.101
Crochetage R wave (*n*, %)	105 (39.3)	14 (21.2)	91 (45.3)	<0.001
RBBB (*n*, %)	30 (11.2)	6 (9.1)	24 (11.9)	0.525
IRBBB (*n*, %)	18 (6.7)	3 (4.5)	15 (7.5)	0.574
CRBBB (*n*, %)	12 (4.4)	3 (4.5)	9 (4.5)	1.000
Crochetage R wave or RBBB (*n*, %)	118 (44.2)	16 (24.2)	102 (50.7)	<0.001
HR (beats/min)	71.0 (62.2, 78.7)	75.0 (64.0, 80.5)	70.0 (62.0, 78.0)	0.061
PFO‐RLS class (*n*, %)	**‐**	**‐**	**‐**	<0.001
Negative	66 (24.7)	66 (100.0)	‐	
Mild	39 (14.6)	‐	39 (19.4)	
Moderate	84 (31.5)	‐	84 (41.8)	
Extensive	78 (29.2)	‐	78 (38.8)	
Echocardiography				
LAD (mm)	36.1 (33.3, 40.3)	37.0 (34.0, 43.0)	36.0 (33.0, 39.0)	0.064
RAD (mm)	31.5 (29.3, 33.9)	32.0 (30.0, 34.0)	32.0 (29.0, 34.0)	0.833
LVD (mm)	45.8 (42.9, 48.2)	46.0 (43.0, 49.0)	46.0 (43.0, 48.0)	0.495
RVD (mm)	23.5 (21.5, 25.9)	24.0 (21.8, 26.0)	23.0 (21.8, 25.0)	0.331
LVEF (%)	63.4 (60.9, 65.5)	63.0 (61.0, 65.0)	64.0 (61.0, 66.0)	0.556
LVFS (%)	34.0 (32.2, 35.8)	34.0 (32.0, 35.0)	34.0 (32.0, 36.0)	0.342

*Note*: Results are expressed as median (interquartile range) or *n* (%).

Abbreviations: BMI, body mass index; CRBBB, complete RBBB; DBP, diastolic blood pressure; HR, heart rate; IRBBB, incomplete RBBB; LAD, left atrial longitudinal diameter; LVD, left ventricular end‐diastolic diameter; LVEF, left ventricular ejection fraction; LVFS, left ventricular fractional shortening; PFO, patent foramen ovale; RAD, right atrial diameter; RBBB, right bundle branch block; RLS, right to left shunt; RVD, right ventricular end‐diastolic diameter; SBP, systolic blood pressure.

### 3.2 The predictive value of TTE and abnormal ECG for PFO

The diagnostic characteristics of TTE, crochetage R wave, and RBBB are shown in Table 2. The accuracies of TTE, crochetage R wave, and RBBB were 0.637 (95% CI: 0.576–0.694), 0.535 (95% CI: 0.474–0.597), and 0.314 (95% CI: 0.259–0.374), respectively. The sensitivity of crochetage R wave was 0.453 (95% CI: 0.383–0.524) and the specificity was 0.788 (95% CI: 0.670–0.879). The results showed that RBBB should not be used to diagnose PFO. But RBBB may improve the pridictive value of crochetage R wave. A combination of crochetage R wave and RBBB demonstrated an accuracy of 0.569 (95% CI: 0.507–0.629), sensitivity of 0.507 (95% CI: 0.436–0.578), and specificity of 0.758 (95% CI: 0.636–0.854). When TTE, crochetage R wave, and RBBB were combined, the accuracy, sensitivity, specificity, PPV, and NPV were 0.712 (95% CI: 0.653–0.765), 0.801 (95% CI: 0.739–0.854), 0.439 (95% CI: 0.317–0.567), 0.813 (95% CI: 0.752–0.865), and 0.42 (95% CI: 0.302–0.545), respectively. Logistic regression analysis revealed a significant correlation between PFO and the presence of crochetage R wave (odds ratio [OR]: 3.073, 95% CI: 1.601–5.899, *p* < 0.001) and also a combination between crochetage R wave and RBBB (OR: 3.220, 95% CI: 1.720–6.028, *p* < 0.001), (Table 3).

**Table 2:** Diagnostic test characteristics of transthoracic echocardiography, right bundle branch block, and crochetage R wave in inferior limb leads in diagnosing patent foramen ovale.

Variables	Accuracy	95% CI	Sensitivity	95% CI	Specificity	95% CI	PPV	95% CI	NPV	95% CI
TTE	0.637	0.576–0.694	0.662	0.592–0.727	0.561	0.433–0.683	0.821	0.753–0.877	0.352	0.262–0.452
Crochetage R wave	0.535	0.474–0.597	0.453	0.383–0.524	0.788	0.670–0.879	0.867	0.786–0.925	0.321	0.250–0.399
RBBB	0.314	0.259–0.374	0.119	0.078–0.172	0.909	0.812–0.966	0.800	0.614–0.923	0.253	0.199–0.314
Crochetage R wave or RBBB	0.569	0.507–0.629	0.507	0.436–0.578	0.758	0.636–0.854	0.864	0.789–0.920	0.336	0.260–0.417
TTE or Crochetage R wave	0.697	0.638–0.751	0.771	0.707–0.827	0.470	0.346–0.597	0.816	0.753–0.868	0.403	0.292–0.521
TTE or Crochetage R wave or RBBB	0.712	0.653–0.765	0.801	0.739–0.854	0.439	0.317–0.567	0.813	0.752–0.865	0.420	0.302–0.545

Abbreviations: CI, confidence interval; NPV, negative predictive value; PPV, positive predictive value; RBBB, right bundle branch block; TTE, transthoracic echocardiography.

**Table 3:** Relationship between the presence of patent foramen ovale and transthoracic echocardiography and the findings of electrocardiogram in univariate logistic regression analysis.

Variables	Odds ratio	95% CI	*p* Value
Crochetage R wave	3.073	1.601–5.899	<0.001
RBBB	1.356	0.529–3.476	0.526
Crochetage R wave or RBBB	3.220	1.720–6.028	<0.001

Abbreviations: CI, confidence interval; RBBB, right bundle branch block; TTE, transthoracic echocardiography.

## 4 DISCUSSION

PFO has been associated with left circulation thromboembolism to several organs.^2^, ^15^ Early diagnosis of PFO is thus very important. ECG is a low‐cost testing method. Crochetage R wave might appear in lead II, III, or avF alone, or in two of them, or in all of them. We found that the ratio of crochetage R wave in patients with PFO was higher than in patients without PFO (45.3% vs. 21.2%). Diagnosing PFO with crochetage R wave had a sensitivity of 45.3% and a specificity of 78.8%. Combining TTE and crochetage R wave and RBBB increased the accuracy. Thus, crochetage R wave in inferior leads may be a good indicator for PFO if a screening test for diagnosing PFO was performed.

The presence of an R wave with a notch in inferior leads was related to PFO. A study by Ay et al.,^12^ including 60 patients with first‐ever stroke or TIA (28 with echo‐documented PFO), showed that crochetage R wave was present more frequently in PFO patients (36% vs. 9%), and the sensitivity and specificity for diagnosis of PFO in cryptogenic stroke cases were 36% and 91%, respectively.

Usually, TTE is the first diagnostic step for detecting PFO. But its sensitivity is low (46%).^3^ In our study, the sensitivity and specificity of TTE were 66.2% (95% CI: 59.2–72.7) and 56.1% (95% CI: 43.3–68.3%), respectively. We believe this may be related to the experience of a sonographer. An experienced sonographer may be able to discover more clues related to PFO, such as RLS appearing with inhalation. TEE with the addition of ASC is considered the gold‐standard imaging modality for identification of PFO, but it is not easily accepted by patients because of its invasiveness and lack of convenience.

PFO and atrial septal defect (ASD) are both direct communications between the two atrial chambers, which allows shunting of blood between the systemic and pulmonary circulations. But a PFO is not considered a true ASD because of no structural deficiency of the atrial septal tissue.^8^, ^16^ The PFO is a “flap” that fails to close after birth, whereas an ASD represents a true defect in the interatrial wall between the right and left atrium.^16^ In the DEFENSE‐PFO study,^17^ 41.7%–43.3% of the patients with PFO with no shunt during rest, 51.7%–56.7% left to right shunt, 5% RLS, and 1.7% bidirectional shunt, respectively. A PFO can be responsible for an intermittent transient or persistent RLS, which is considered to be related to an unexplained left circulation embolism when right atrial pressure exceeds left atrial pressure (i.e., cough, Valsalva maneuver, volume overload, positive end‐expiratory pressure, pulmonary vascular disease). PFO is thought to cause about 50% of cryptogenic strokes.^3^ European position paper^2^ figured out that the event should be classified as PFO‐related instead of cryptogenic when a PFO was thought likely to be implicated in a cryptogenic embolism. A meta‐analysis of case–control studies showed a significant association between PFO and ischemic stroke in patients <55 years old (OR: 3.1, 95% CI: 2.29–4.21).^18^ A multivariate analysis of a prospective case–control study of 503 consecutive patients with ischemic stroke showed that the presence of a PFO was independently associated with cryptogenic stroke both in patients <55 years old (OR: 3.7, 95% CI: 1.42–9.65) and in patients ≥55 years old (OR: 3.0, 95% CI: 1.73–5.23).^19^ The Risk of Paradoxical Embolism (RoPE) study,^20^ which was a meta‐analysis of 12 cryptogenic stroke cohorts, found a correlation between the prevalence of PFO and the likelihood that PFO was the underlying etiology of stroke without vascular risk factors such as hypertension, diabetes, smoking, and prior transient ischemic attack and the presence of a cortical infarct. A cohort study^21^ found that RLS induced by a PFO was associated with an increased risk of cryptogenic stroke. The study^21^ showed a rising trend in the proportion of small lesions and the influenced posterior circulation with an increasing RLS in different cryptogenic stroke patients. Otherwise, PFO was associated with decompression sickness,^4^ secondary migraine headache,^5^ arterial deoxygenation, and platypnea‐orthodeoxia.^6^, ^7^ European position paper^6^ suggested proposing PFO closure appropriately.

Crochetage R wave was related to ASD in previous studies. In a study by Heller et al.,^11^ including a total of 532 patients with ASD compared to those of healthy individuals in the control group, crochetage R wave was observed in 73.1% of ASD patients in one lead at least, 58.1% in two to three leads, and 27.8% in three leads. The sensitivity and specificity of crochetage R wave for the diagnosis of ASD were reported to be 73.1% and 92.6%, respectively.^11^ It was reported that the specificity was 100% when it was present in three leads.^11^ A cohort study,^22^ which included 256 children with secundum ASD and 256 age‐ and gender‐matched children without heart disease, showed that the incidence of crochetage R wave in all three inferior limb leads was 28.13% of subjects with secundum ASD, and it was only 2.73% of subjects with secundum ASD. A retrospective study,^23^ which included a total of 314 patients who underwent percutaneous device closure for secundum ASD, showed that crochetage R wave was an independent predictor for late atrial fibrillation or atrial flutter (hazard ratio: 3.90, 95% CI: 2.05–7.76, *p* < 0.001). Thus, crochetage R wave may also predict atrial fibrillation or atrial flutter in patients who underwent PFO closure.

In addition to crochetage R wave, RBBB was considered as a marker for ASD. It was reported that the observed rsR′ or rSr′ patterns in patients with ASD are related to right ventricular overload, rather than true propagation delay.^24^ A prospective study^10^ included 87 adult patients with suspicion of ASD, showing that PFO was significantly more prevalent in patients with RBBB (39.02% vs. 4.3%, *p* < 0.001). Another study^25^ included 61 patients diagnosed with ASD, showing that incomplete RBBB (56% vs. 5%) was more frequent in ASD patients compared to the control group patients and was related to ASD (OR: 26, 95% CI: 7–94). The possible mechanisms responsible for this relationship between RBBB and ASD may be: abnormal delay in right ventricular activation due to enlarged right ventricular cavity size; progressive, chronic endocardial fibrosis, caused by turbulence in the interruption region of atrial septum that could directly damage the conduction system; right ventricular overload and stretch of the conduction system.^10^ However, there was no significant association between RBBB and PFO in our study. Although we were unable to explain precisely the relationship, combined crochetage R wave and RBBB might improve the accuracy for the diagnosis of PFO.

Otherwise, defective T wave (DTW) of ECG was considered as a marker for ASD. DTW is a double‐peaked T wave with a prominent second summit, the delayed Q–T peak interval in V2 compared with the interval in V6. It has been reported in a study that DTW alone had 87.9% sensitivity and 97.0% specificity for the diagnosis of ASD, and coexistence of incomplete RBBB and DTW had 87.1% sensitivity and 100% specificity.^26^ Another study showed 48% sensitivity and 97% specificity of DTW alone and 36% sensitivity and 100% specificity of coexistence of incomplete RBBB and DTW for the diagnosis of ASD.^25^ However, there were only two cases with DTW in our study, and RBBB simultaneously present. We did not analyze the data of DTW for the diagnosis of PFO.

In particular, a combination of TTE and crochetage R wave and RBBB has 80.1% sensitivity and 43.9% specificity in our study. However, TEE with ASC echocardiography cannot be replaced by ECG and TTE. Therefore, a patient with a PFO may be diagnosed by TTE, or crochetage R wave or RBBB in ECG should be suggested to undergo TEE with ASC echocardiography to identify the real PFO.

## 5 LIMITATIONS

Our study was a retrospective study using a continuous inclusion of patients who had undergone ECG, TTE, TEE, and ASC echocardiography in our hospital, ages and genders were not matched, and the incidence of PFO was not the true incidence in the general population.

## 6 CONCLUSION

Crochetage R wave in ECG is associated with PFO. Crochetage R wave, especially combined with RBBB and TTE, may be helpful in the early detection of patients with PFO. Our study suggested that a patient with crochetage R wave in ECG should undergo TEE with ASC echocardiography to identify PFO.

## CONFLICT OF INTEREST STATEMENT

The authors declare no conflict of interest.

## Data Availability

The data that support the findings of this study are available from the corresponding author upon reasonable request.
